# 1748. Epidemiology and resistance patterns of nursing home uropathogens in four states, 2018-2019

**DOI:** 10.1093/ofid/ofac492.1378

**Published:** 2022-12-15

**Authors:** Lindsay Taylor, Jessica Irvine, Sally Jolles, Taissa A Bej, Corinne Kowal, Raymond Podzorski, Robin L Jump, Christopher J Crnich

**Affiliations:** University of Wisconsin School of Medicine and Public Health, Madison, Wisconsin; University of Wisconsin School of Medicine and Public Health, Madison, Wisconsin; University of Wisconsin School of Medicine and Public Health, Madison, Wisconsin; Louis Stokes Cleveland VA Medical Center, Cleveland, Ohio; Louis Stokes Cleveland VA Medical Center, Cleveland, Ohio; St. Mary's Hospital Laboratory, Madison, Wisconsin; University of Pittsburgh School of Medicine, Cleveland, Ohio; University of Wisconsin School of Medicine and Public Health, Madison, Wisconsin

## Abstract

**Background:**

Urinary tract infections (UTIs) are the most common indication for antibiotic treatment in nursing homes (NHs) and are often treated empirically before culture results return. A previous study of uropathogens from NH residents in Georgia revealed high resistance to commonly used antibiotics, including ciprofloxacin. Here, we describe the distribution and key resistance patterns of uropathogens recovered from residents of NHs in Wisconsin, Ohio, Kentucky, and Indiana.

**Methods:**

Results of urinary cultures from NH residents performed between 2018 and 2019 were obtained from contracting microbiology laboratories. Culture results were de-identified by an honest broker. Urine culture data was standardized across laboratories and years using key collision and nearest neighbor clustering methods. Non-pathogenic isolates were excluded. The 5 most common isolates were identified and mean susceptibilities to antibiotics commonly used for empiric treatment of UTIs were examined. Data standardization and analysis were performed in Open Refine and R.

**Results:**

There were 11,007 urine cultures obtained from 5,642 NH residents living in 67 NHs. A total of 4,398 potentially uropathogens were identified (avg. per NH = 66; range = 2-279). *Escherichia coli* (43.1%), *Klebsiella* spp. (14.6%), *Proteus* spp. (14.3%), *Enterococcus* spp. (9.5%), and *Pseudomonas* spp. (4.6%) were the most commonly identified uropathogens. Resistance rates for the majority of evaluated bug-drug combinations (9/14) exceeded 20% (Figure 1). Nitrofurantoin was the only antibiotic with a resistance rate < 20% for *E. coli* isolates. When resistance was assessed cumulatively across the top 5 organism, 48.5% of isolates were resistant to ciprofloxacin and 21.3% of isolates were resistant to ceftriaxone (when excluding isolates with intrinsic resistance).

Percentage of common urinary isolates resistant to most common antibiotics

**Figure:**
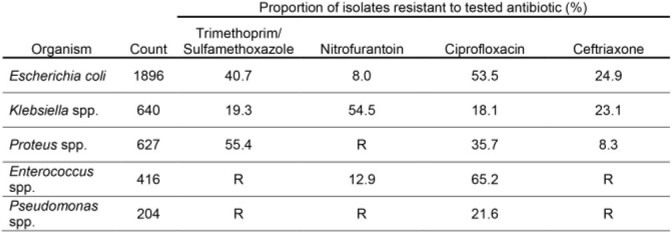

R denotes intrinsic resistance. Abbreviation: species pluralis = spp.

**Conclusion:**

Consistent with a recent study conducted in Georgia NHs, high levels of resistance to commonly used antibiotics amongst uropathogens recovered from NH residents in four states were identified in the current study. These suggest a need for NH-specific UTI treatment guidelines and tools to support empiric antibiotic decision-making at the local facility level.

**Disclosures:**

**Robin L. Jump, MD, PhD**, Merck: Grant/Research Support|Pfizer: Advisor/Consultant.

